# Supplementary figures and images for: Gammaretroviral vector encoding a fluorescent marker to facilitate detection of reprogrammed human fibroblasts during iPSC generation
Source: PeerJ. 2013 Dec 10;1:e224. doi: 10.7717/peerj.224 (PMC3869187; doi:10.7717/peerj.224)

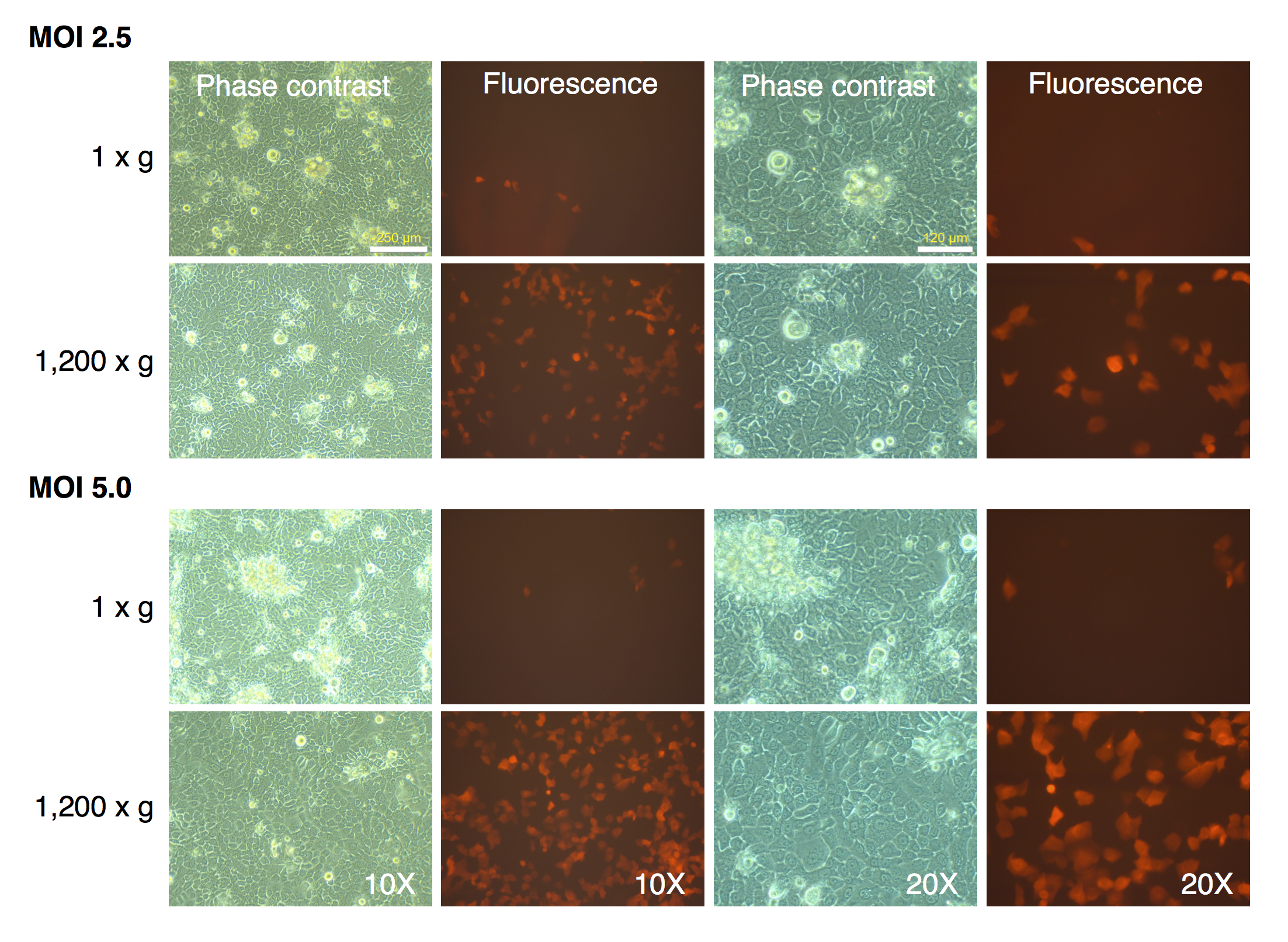

Supplement: Figure S1 — Primary human keratinocytes (Invitrogen Corporation, USA, Catalog number 12332-001) were transduced with pMXs-mRFP1 vector at unit gravity (1 × g) or by spin-transduction (1,200 × g for 2 h at room temperature). Two different MOIs were used (2.5 and 5.0). The cells were visualized by phase contrast and fluorescence microscopy. [file peerj-01-224-s001.jpg]

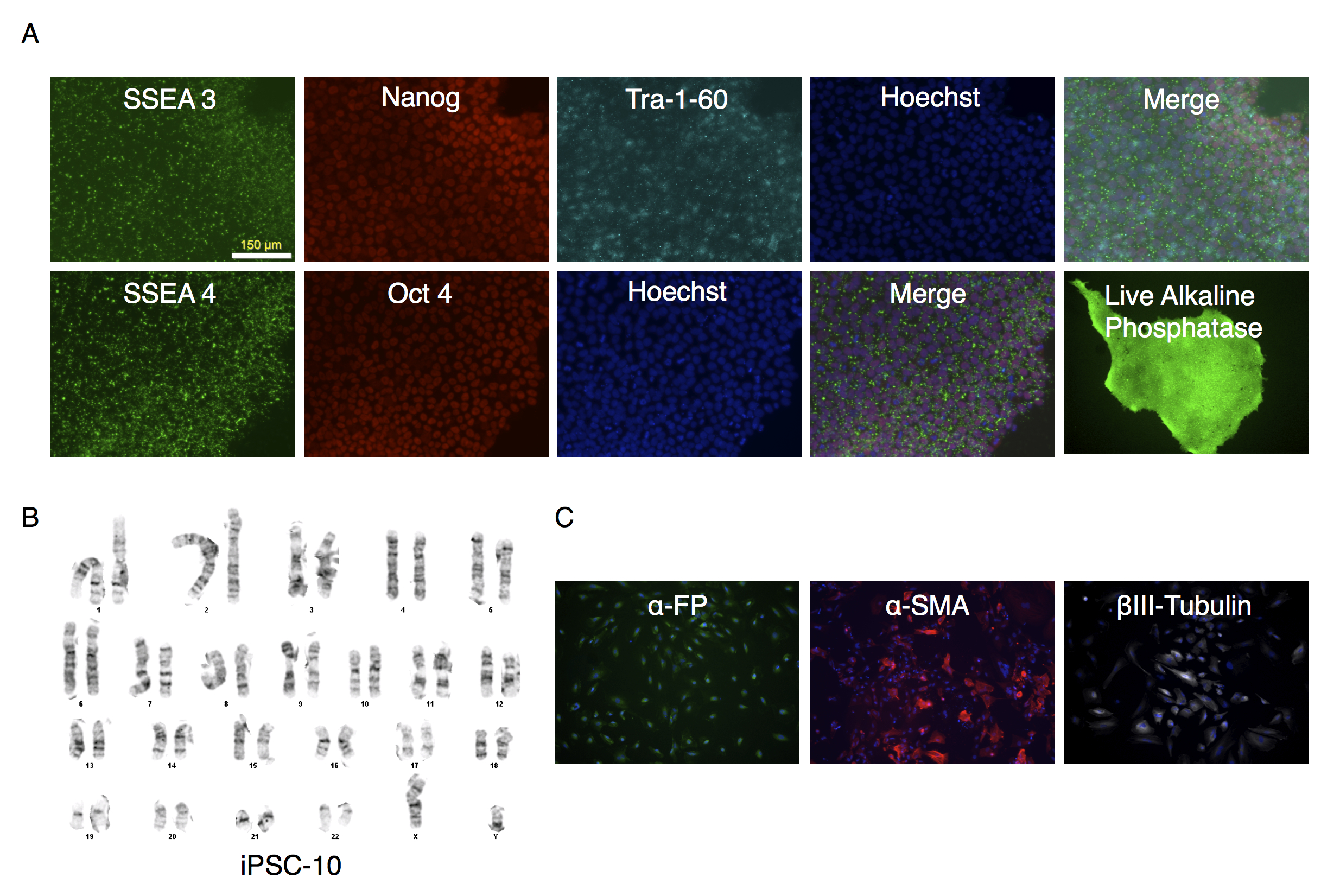

Supplement: Figure S2 — (A) Parallel wells of iPSC-10 clone were fixed with paraformaldehyde and stained with antibodies to SSEA4, Nanog, Tra-1-60 or SSEA-3 and Oct3/4. Nuclei were stained using Hoechst 33342. Alkaline phosphatase expression was determined on unfixed cultures as described in the text. The stained colonies were visualized under fluorescence microscopy. (B) Karyotype analysis of clone. (C) Embryoid bodies were allowed to attach and spread out on gelatin-coated wells, fixed and stained for endodermal (α-fetoprotein (α-FP)), mesodermal (α-smooth muscle actin (α-SMA)), or ectodermal (βIII-Tubulin) markers. [file peerj-01-224-s002.jpg]

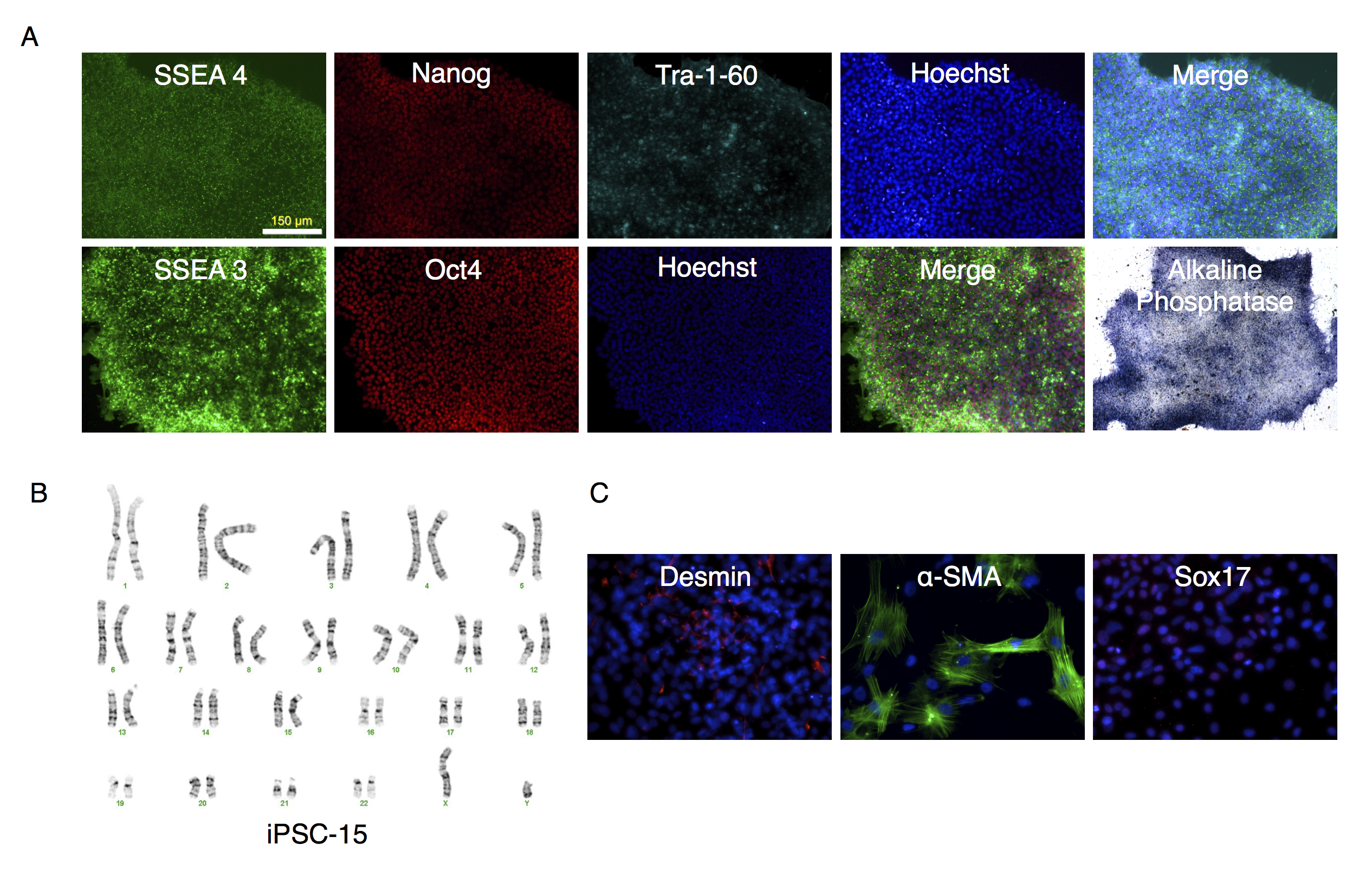

Supplement: Figure S3 — (A) Parallel wells of iPSC-15 clone were fixed with paraformaldehyde and stained with antibodies to SSEA4, Nanog, Tra-1-60 or SSEA-3 and Oct3/4 as described in the text. Nuclei were stained using Hoechst 33342. The stained colonies were visualized under fluorescence microscopy. The alkaline phosphatase stained colonies were visualized under bright field microscopy. (B) Karyotype analysis of clone. (C) Embryoid bodies were allowed to attach and spread out on gelatin-coated wells, fixed and stained for desmin, α-smooth muscle actin (α-SMA ) actin, or Sox17 differentiation markers. [file peerj-01-224-s003.jpg]

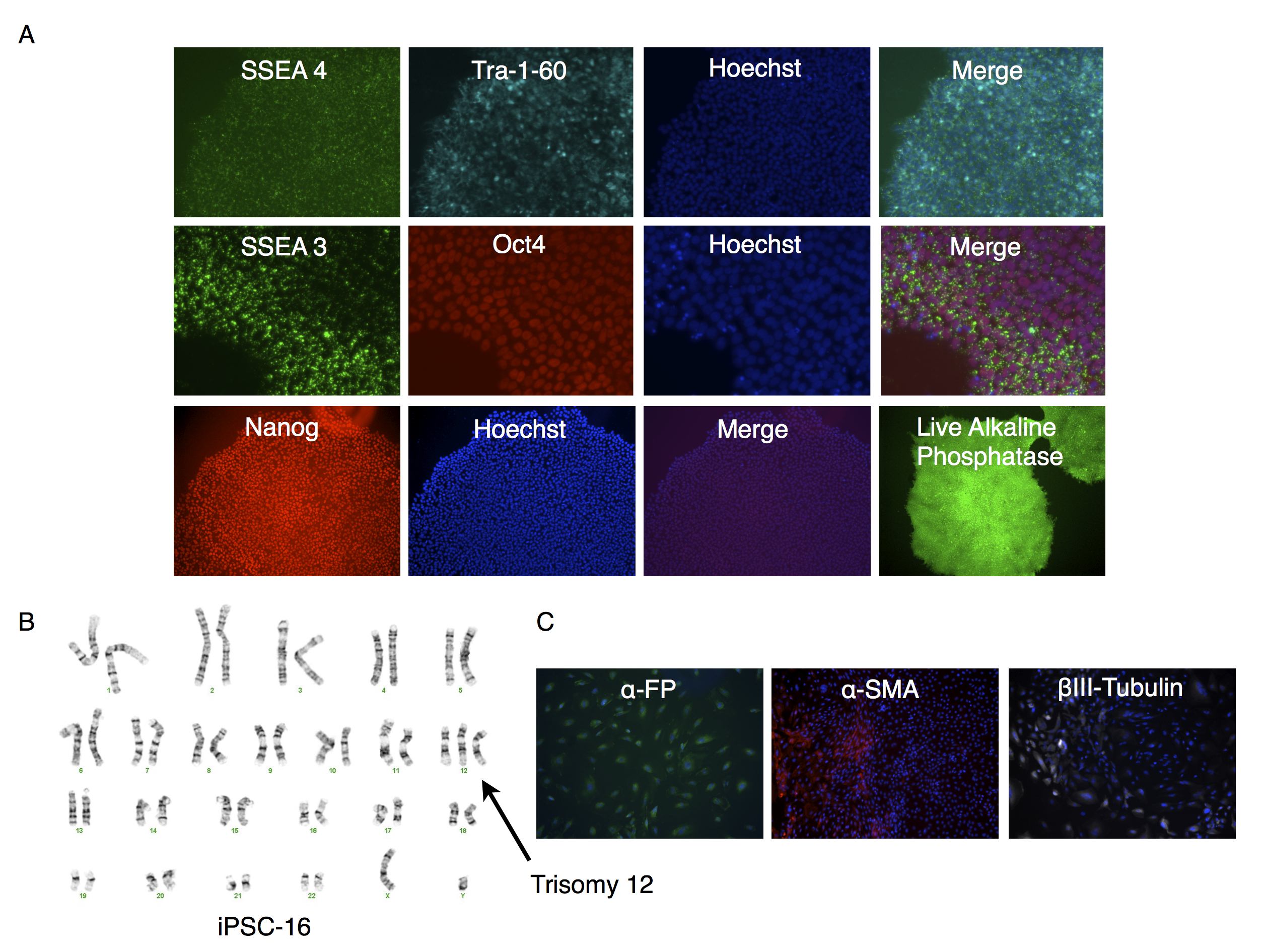

Supplement: Figure S4 — (A) Parallel wells of iPSC-16 clone were fixed with paraformaldehyde and stained with antibodies to SSEA4, Tra-1-60 or SSEA-3 and Oct3/4 or Nanog and visualized under fluorescence microscopy. Nuclei were stained using Hoechst 33342 (top two rows of photomicrographs) or Dapi (for Nanog counter stain). Alkaline phosphatase expression was determined on unfixed cultures as described in the text. (B) Karyotype analysis of clone. Arrow points to trisomy of chromosome 12. (C) Embryoid bodies were allowed to attach and spread out on gelatin coated wells, fixed and stained for endodermal (α-fetoprotein (α-FP)), mesodermal (α-smooth muscle actin (α-SMA)), or ectodermal (βIII-Tubulin) markers. [file peerj-01-224-s004.jpg]

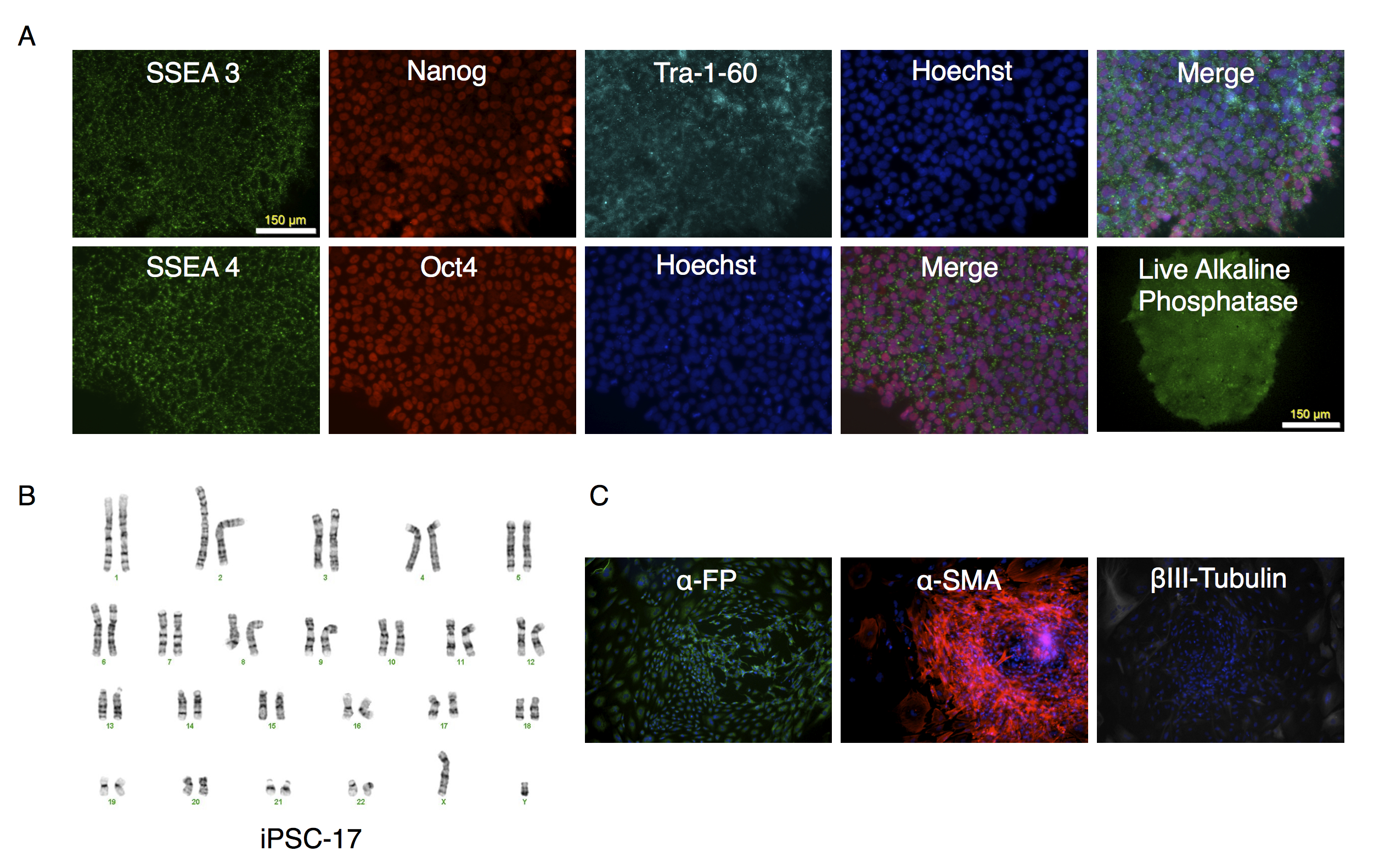

Supplement: Figure S5 — (A) Parallel wells of iPSC-17 clone were fixed with paraformaldehyde and stained with antibodies to SSEA3, Nanog, Tra-1-60 or SSEA4 and Oct3/4. Nuclei were stained using Hoechst 33342. Alkaline phosphatase expression was determined on unfixed cultures as described in the text. The stained colonies were visualized under fluorescence microscopy. (B) Karyotype analysis of clone. (C) Embryoid bodies were allowed to attach and spread out on gelatin coated wells, fixed and stained for endodermal (α-fetoprotein (α-FP)), mesodermal (α-smooth muscle actin (α-SMA)), or ectodermal (βIII-Tubulin) markers. [file peerj-01-224-s005.jpg]

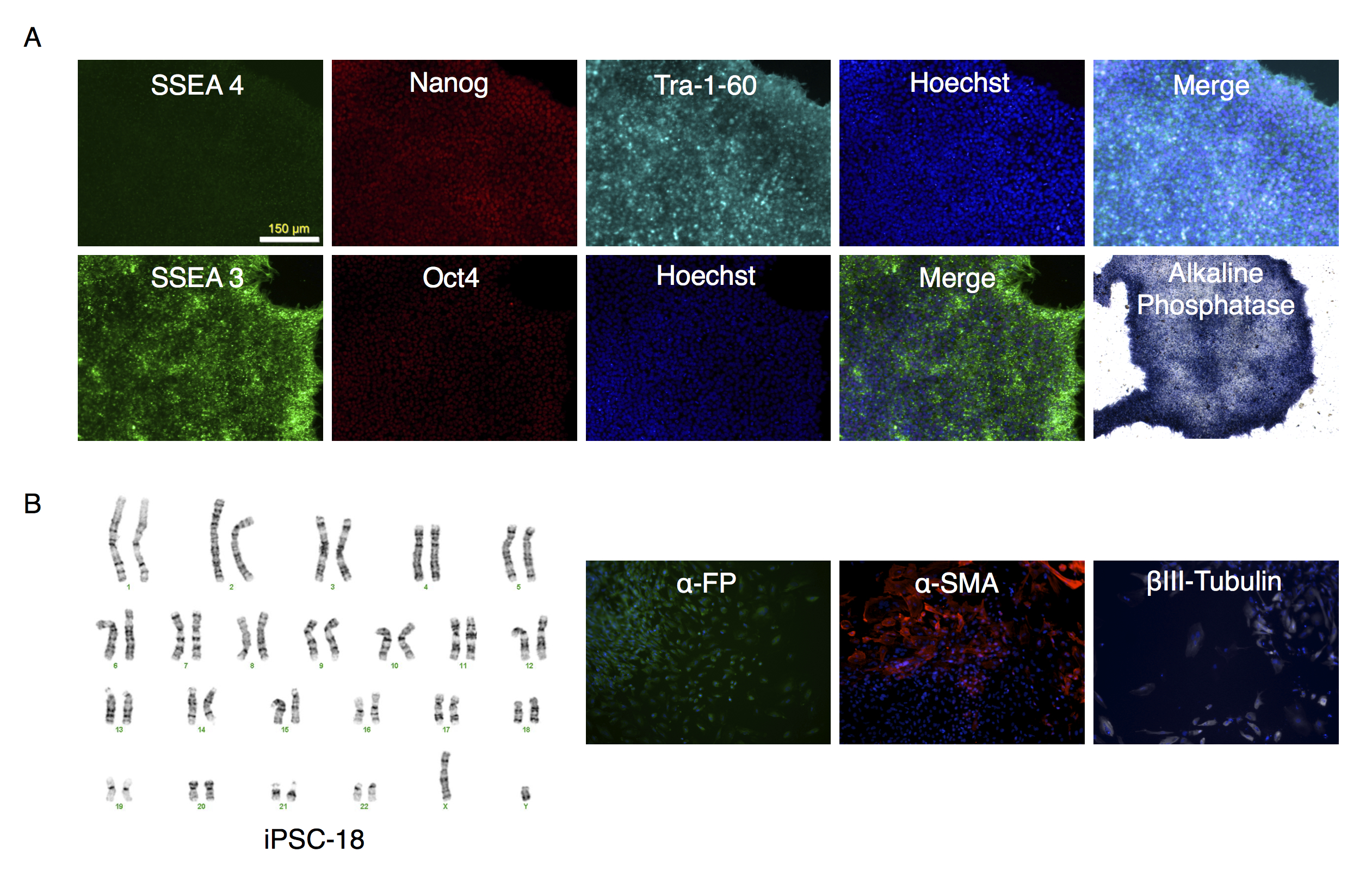

Supplement: Figure S6 — (A) Parallel wells of iPSC-18 clone were fixed with paraformaldehyde and stained with SSEA4, Nanog and Tra-1-60, or SSEA-3 and Oct3/4. Nuclei were stained with Hoechst. The colonies were visualized under fluorescence microscopy. The alkaline phosphatase stained colonies were visualized under bright field microscopy. (B) Karyotype of clone. (C) Embryoid bodies were allowed to attach and spread out on gelatin coated wells, fixed and stained for endodermal (α-fetoprotein (α-FP)), mesodermal (α-smooth muscle actin (α-SMA)), or ectodermal (βIII-Tubulin) markers. [file peerj-01-224-s006.jpg]

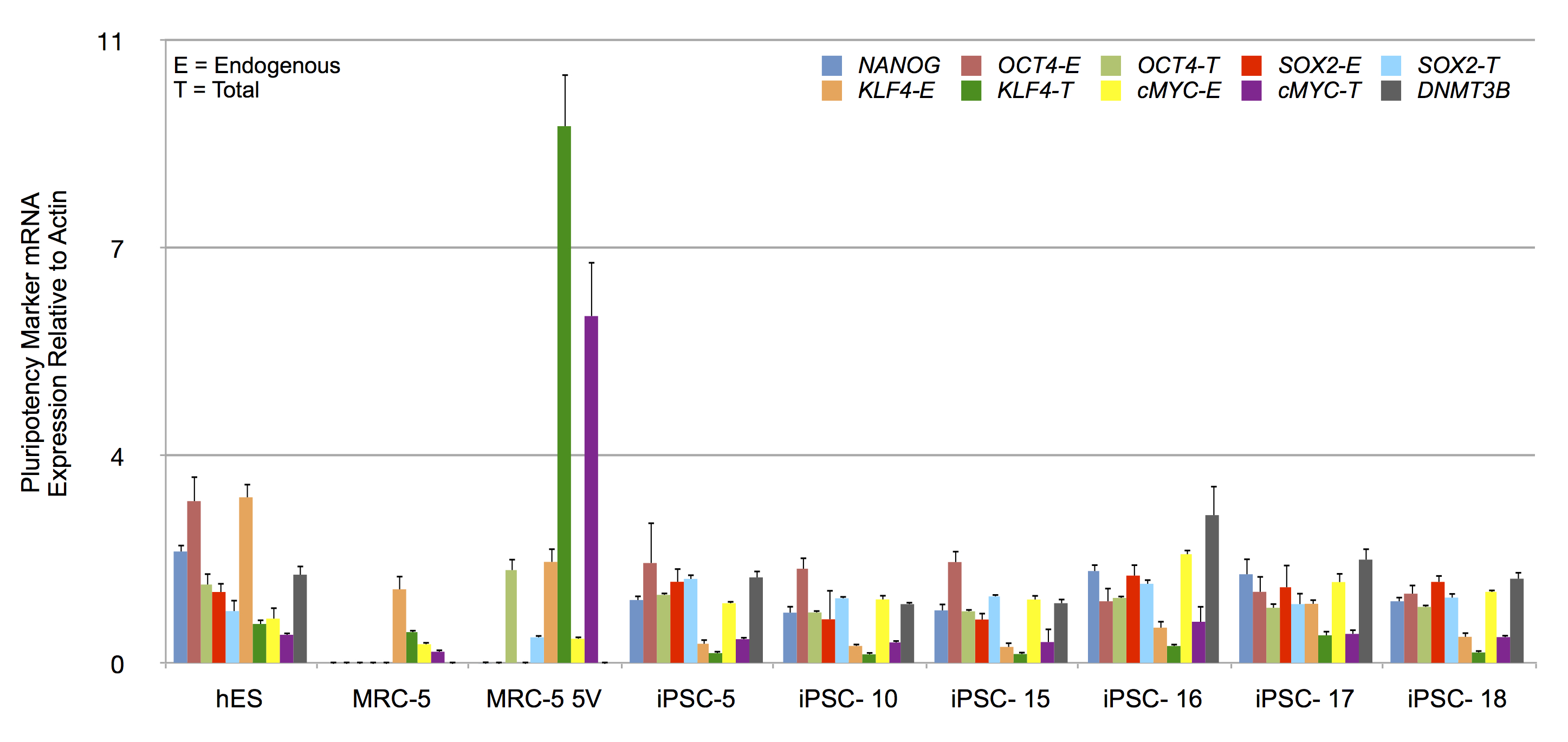

Supplement: Figure S7 — Expression of indicated plutipotency markers (NANOG), DNA methyltransferase 3B (DNMT3B) and RFs (OCT4, SOX2, KLF4 and cMYC) in hES cells, untransduced MRC-5 and transduced MRC-5 (MRC-5 5V), and derived iPSC clones. The mRNA expression was normalized to β-actin levels in the samples. Error bar represents one standard deviation. The standard deviation of the ratio of means was calculated as described under Materials and Methods. [file peerj-01-224-s007.jpg]

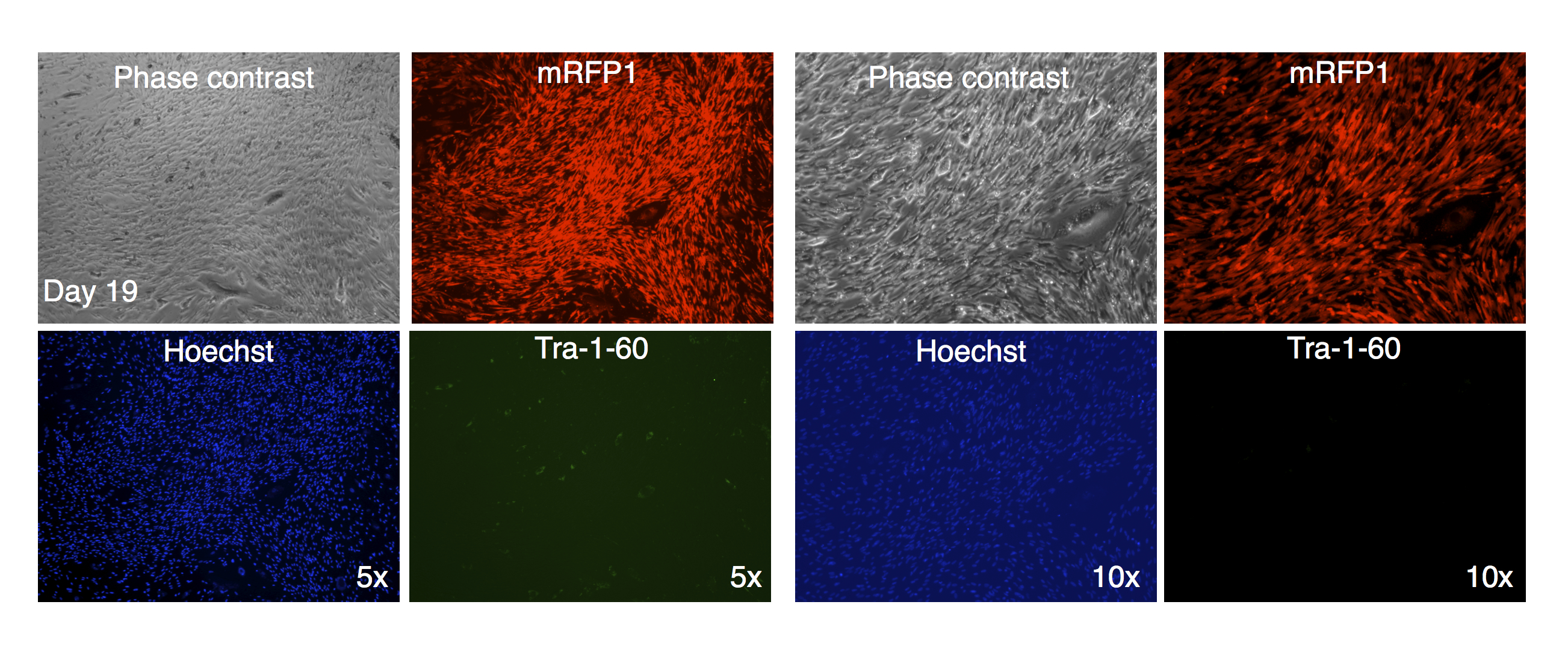

Supplement: Figure S8 — An mRFP1-positive cellular aggregate in the same well that contained the mRFP1-negative colony (Fig. 11) was visualized under phase and fluorescence microscopy as described in the text and legend to Fig. 11. [file peerj-01-224-s008.jpg]
